# Career factors related to winning Olympic medals in swimming

**DOI:** 10.1371/journal.pone.0304444

**Published:** 2024-06-28

**Authors:** Aslan Tchamkerten, Paul Chaudron, Nicolas Girard, Antoine Monnier, David B. Pyne, Philippe Hellard

**Affiliations:** 1 Department of Communications and Electronics, Institute Polytechnique de Paris, Telecom Paris, Paris, France; 2 Research Institute for Sport and Exercise, University of Canberra, Canberra, Australia; 3 CREPS Resource and Expertise Center on Sports Performance in Bordeaux, Bordeaux, France; 4 CETAPS EA3832, Rouen University, Rouen, France; Kokushikan University, JAPAN

## Abstract

To investigate associations between a swimmer’s career progression and winning a medal at the Olympic Games (OG) or World Championships (WC). A total of 4631 swimming performances of 1535 top swimmers (653 women, 882 men) from 105 nationalities since1973 were extracted from FINA rankings. A panel of 12 predictor variables including nationality, gender, competition, age, number and timing of competitions, pattern of progressions and regressions in performance, and medal outcomes was established. Linear logistic regression was used to study the association between winning a medal and predictor variables. Logistic regression coefficients were obtained by training on 80% of the database, and prediction accuracy evaluated on the remaining 20%. Using the training set, a selection of 9 most relevant features for prediction of winning a medal (target variable) was obtained through exhaustive feature selection and cross-validation: nationality, competition, number of competitions, number of annual career progressions (nb_prog), maximum annual career progression (max-progr), number of annual career regressions (nb_reg), age at maximum annual progression, P6 (the level of performance six months before the World Championships or Olympic Games), and P2 (the level of performance two months before the World Championships or Olympic Games). A logistic regression model was built and retrained on the entire training set achieved an area under the ROC curve of ~90% on the test set. The odds of winning a medal increased by 1.64 (95% CI, 1.39–1.91) and 1.44 (1.22–1.72) for each unit of increase in max-progr and n-prog, respectively. Odds of winning a medal decreased by 0.60 (0.49–0.72) for a unit increase in n-reg. In contrast, the odds increased by 1.70 (1.39–2.07) and 4.35 (3.48–5.42) for improvements in the 6 and 2 months before competition (P<0.001, for all variables). The likelihood of a swimmer winning an international medal is improved by ~40–90% with progressions from season-to-season, and reducing the number of regressions in performance. The chances of success are also improved 2- to 4-fold by substantial improvements in performance in the months before competition.

## Introduction

Analyzing the age-related progression of elite athletes to their career-best performances allows us to identify factors that lead to success, and predict the results of international sporting competitions [1–6]. Winning medals at the level of the Olympic Games or World Championships is the result of a multidimensional process in which multiple factors interact throughout the athletes’ careers [4–7] depends on genetics [8], social factors such as support from families, coaches, peers and support staff [9], elite performance environments [10], and optimized training programs [11,12].

Analysis of long-term career changes in performance using past competition results is a useful approach for understanding and predicting athletic success at the world level [1–6,13]. This type of analysis has been applied in track-and-field athletics [4,5,14–16], swimming [1–3], triathlon [13] and cross-country skiing [5]. Several research projects on the subject [4–6,14,15] modeled performance changes and estimated the age and level of performance peaks over time. The typical empirical pattern of performance evolution is composed of an exponential acceleration during the pubertal developmental period which leads to a peak in performance sustained in a plateau over 1–4 years [4,5,14–16]. However, population-wide patterns do not account for heterogeneity and intra-individual fluctuations [4–6,15,16]. Indeed, analysis of individual trajectories highlights broad heterogeneity with oscillations that represent multiple periods of micro progressions and regressions of annual individual best performances or individual positions in world rankings throughout the career of athletes [14–17], triathletes [13] and swimmers [1,18].

Performance progressions can differentiate an athlete’s level in the post-puberty phase that extends from ~14 years for girls and ~16 years for boys. Work in swimming [1–3,19], track and field [4,17] and cross-country skiing [5] have shown a higher rate of performance acceleration (i.e. a higher progression in performance level or individual position in world rankings) for world-class athletes than non-world-class athletes and for athletes ranked in the world’s top 10 compared to those ranked 11 to 100 [1–5,19]. On the shorter scale of the Olympic year, the progression of performances over the season increases the chances of winning a medal [20,21]. The majority (87%) of the Olympic medalists were world top 10 ranked before the Olympic Games [20], Olympic medal swimmers showed a mean performance increase of ~1% during the Olympic year, and medalists and finalists were able to improve on their previous season’s best performances during the major events [21,22].

Analysis of long-term changes in swimmer performance can be used to predict peak performance and performance plateaus, the number of qualified swimmers, and performances at the Olympics [1,2]. Although Bullock and Hopkins [23] modeled skeleton race placing by quadratic curves and extrapolating to predicted Olympic outcomes, no study has modeled the probability of winning Olympic and world medals in swimming. One way to estimate this probability is to consider the evolution of the athlete’s individual performances and relate them to the level of global competition. From this perspective, analysis of individual performance trajectories in relation to world rankings permits identification of different variables that shed light on the entire duration of swimmers’ careers, and can improve the understanding of Olympic and world competitive success.

Among the factors associated with world and Olympic success, a progression in performance and world ranking in the pre-Olympic year has been linked to a greater likelihood of winning a world or Olympic medal [19–21]. For American and Australian Olympic-qualified swimmers, performance gains were approximately 1% in the 12 months between the Pan-Pacific Games and the Olympics, 0.6% in the four months between the Australian Olympic trials and the OGs, and 0.2% in the last five weeks between the American Olympic trials and the OGs [19]. In 4 FINA World Championships and 2 Olympic Games between 2011 and 2017 only medal winners (-0.87% [0.91%]) and finalists (-0.16% [0.97%]) managed to progress between the selection trials and the OG [20]. In the latter study by Mujika et al. [20], an improvement in performance between selection trials and the Olympic Games was only observed for American swimmers (-0.44% [1.08%]) and not for those from other nations, suggesting that the culture of Olympic success may be nationality dependent [24] as already evidenced for Norway in the winter disciplines [12], sprinting in Jamaica [25] and sailing in Denmark [26].

Variables such as nationality, age of entry into the world top 100, maximum career progressions and regressions, number of career progressions and regressions, and performance progression during the Olympic season are all potentially explanatory of success at the World Championships and Olympic Games although previous research has not demonstrated these relationships or indicated the most influential variables. Moreover, previous works focus on descriptive inference at the population level as opposed to prediction at the individual level. The aim of this study was to develop a model for accurate predictions of medal attainment from variables related to the evolution of individual performances pathways throughout the swimmers’ careers. This model could identify the most influential factors linked to world and Olympic swimming success, predict medal chances by identifying individual strengths and weaknesses in swimmers’ careers and suggest potential areas for optimization.

## Methods

### Data

Competition performances at WC/OG events in 50-m pools (long course meters) over 26 swim disciplines were collected from the FINA website (https://www.fina.org/ - API: https://api.fina.org/fina/), restricted to swimmers who appeared at least once during their career in the world top 100 of their discipline. For a given event, swim discipline, and swimmer, only the athlete’s best time was considered. The WC/OG events covered 13 men’s events (50, 100, 200, 400, 1500 freestyle, 100, 200 butterfly, 100, 200 backstroke, 100, 200 breastroke, 200, 400 medley) and 13 women’s events (50, 100, 200, 400, 800, freestyle, 100, 200 butterfly, 100, 200 backstroke, 100, 200 breastroke, 200, 400 medley) from 1973 to 2021.

A table with individual performance was built (see supplementary dataset). Each performance was uniquely identified with the name of the athlete, the swim discipline, and the year of the performance. Each performance specified whether the athlete obtained a medal (label = 1) or not (label = 0) at the corresponding event, and listed the following 12 features used as medal predictor variables:

- Nationality: swimmers from the USA US = 0, swimmers of other nationalities non-US = 1.
- Gender: male (m) = 0, female (f) = 1.
- Comp_type (Types of competitions): OG = 0, WC = 1.
- Entry_age: age at which the athlete first entered the world’s top 100 in the swim discipline.
- Nb_comp (Number of competitions): one plus the total number of WC competitions attended before a WC event, or one plus the total number of OG competitions attended before a OG event, since the athlete entered the world’s top 100.
- Nb_prog: the number of ranking progressions, in the years preceding the year of the competition, starting from the year when the athlete entered the world’s top 100, in the swim discipline.
- Max_prog: the greatest ranking progression between two consecutive years in the years preceding the year of the competition, starting from the year when the athlete entered the world’s top 100, in the swim discipline.
- Max_prog_age: age at which the athlete achieved max_prog in the swim discipline.
- Nb_reg: the number of ranking regressions in the years preceding the year of the competition, starting from the year when the athlete entered the world’s top 100, in the swim discipline.
- Max_reg: same as max_prog but for regression.
- P2: ratio of overall best time in the year preceding the year of the competition, and athlete’s best time in the 2-month period prior to the event, in the swim discipline.
- P6: ratio of overall best time in the year preceding the year of the competition and athlete’s best time in the 2 to 6 months period prior to the event, in the swim discipline.

For the features nb_prog, max_prog, nb_reg, max_reg an athlete that exited the top 100 was assigned rank 100. To illustrate how P2 was computed, let us consider the first entry of the table in (see supplementary dataset) which corresponds to Emma McKeon (coded as non-US) who won the 50 m Freestyle Gold medal at the 2020 Tokyo OG. Her best performance two months before the competition (from 05/22/2021 to 07/21/2021) was 23.93 s. The woman’s overall best performance in 50 m Freestyle in 2020 was 24.03 s. The corresponding P2 is thus 24.03/23.93 = 1.0042.

### Missing data

To deal with missing data, only performances with complete data (i.e., for which the 12 features and the value of the target variable could be retrieved from the FINA website) were considered. The resulting table contained 4631 entries (athlete / swim discipline / year) from 1535 athletes (653 women, 882 men) from 105 nationalities over 26 swim events (13 women’s events and 13 men’s events).

### Standardization

Given that the variables had different scales (ages in years, progressions/regressions in number of places, performances measured in seconds) the features were standardized on a variable-by-variable basis (rescaling to get zero mean and unit variance) using https://scikitlearn.org/stable/modules/generated/sklearn.preprocessing.StandardScaler.html.

The entire database is available at the following link https://www.kaggle.com/datasets/paulchaudron/sequential-model-database-swimming-prediction.

### Feature selection and prediction

To examine the dependency between winning a medal at the WC or OG (target variable) and the predictor variables we used a linear logistic regression model with cross-entropy as loss function. The dataset was divided into a training set (80%) and a test set (20%). The training set and the test set were randomly stratified to guarantee the same proportion of medalists in each using https://scikitlearn.org/stable/modules/generated/sklearn.model_selection.train_test_split.html For the training set a selection of the most relevant variables was obtained through exhaustive feature selection and five-fold cross-validation. This process reduced the number of features from 12 to the following 9: nationality, comp_type, nb_comp, max_prog, nb_prog, max_prog_age, nb_reg, P2, and P6. The logistic regression coefficients corresponding to these variables were obtained through a refit on the entire training set. For each of these coefficients and the constant term (intercept), standard error, z-statistic, p-value, and confidence interval (lower 95%, upper 95%) were computed.

## Results

### Basic statistics

**Fig 1:**
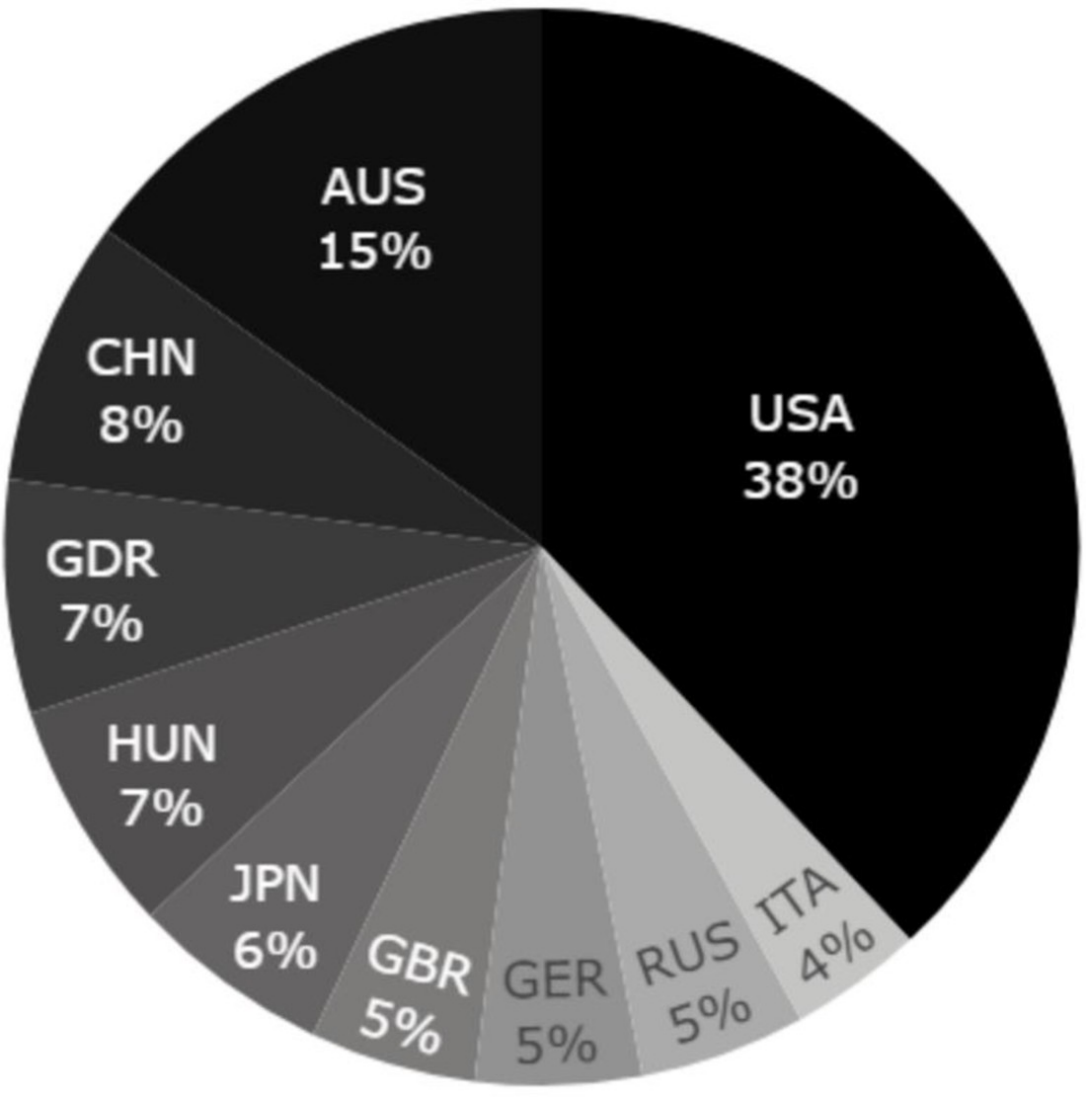
Proportion of medal-winning swimmers by nation in Olympic Games and World Championships 1973–2020 (Olympic Games 1984–2020 and World Championships 1973–2019).

Table 1 shows the proportions of medalists. Only 16.7% of the entire cohort of 1535 swimmers were medalists, 5.3% won gold, 7.2% silver and 6.8% bronze Table 1. Fig 1 shows the proportion of medal winners by nationality.

**Table 1:** Demographic variables. Number and proportion of male and female gold, silver, and bronze medalists in the entire cohort of 1535 swimmers.

	Number	Percentage
**Total number of swimmers**	1535	100
**Men gold medalists**	82	5.3
**Men silver medalists**	111	7.2
**Men bronze medalists**	105	6.8
**Men not medalists**	740	48.2
**Women gold medalists**	79	5.1
**Women silver medalists**	96	6.3
**Women bronze medalists**	95	6.2
**Women not medalists**	538	35.0

**Fig 2:**
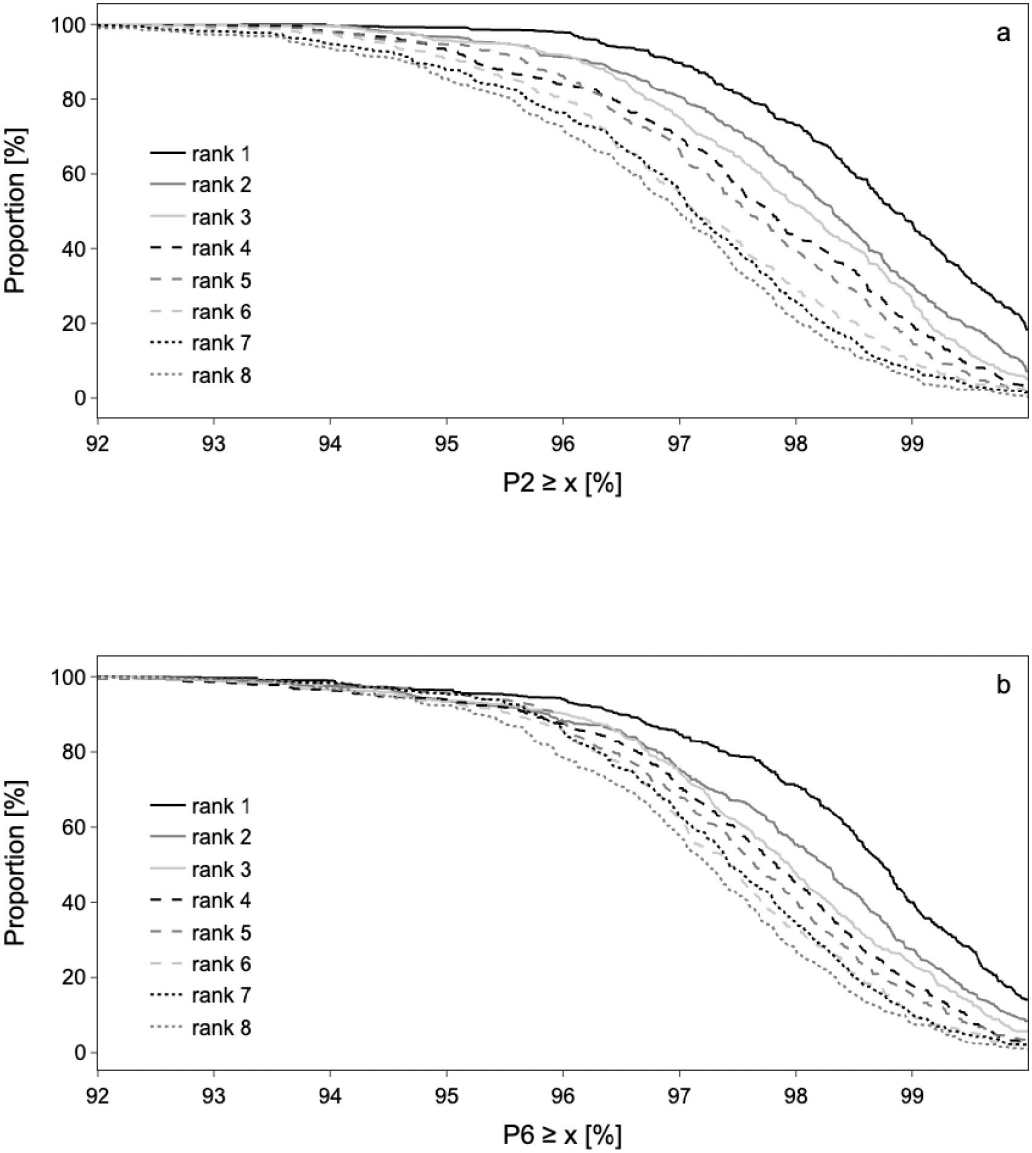
Influence of the variables P2 (a) and P6 (b) on the rank. The rank indicates the ranking at the Olympic Games or World Championships. About 50% of gold medals are associated with a p2 > = 99%. This proportion drops to about 30% for silver medals. P2 indicates the value of the best performance in the two months before the competition (World Championships or Olympic Games) and P6 the best performance in the six months before the competition.

Fig 2a shows the influence of P2 on the final rank at the WC or OG. Each curve represents the proportion of athletes with a given rank as a function of P2, and similarly for Fig 2b with respect to P6. For instance, about 50% of gold medals are associated to a P2 value of at least 99%, and this proportion drops to about 30% for bronze medals. More generally, the better the ranking, the better the performance in the 6- and 2-months windows leading up to the World Championships or Olympics relative to the previous year’s best performance.

### Predictive performance

**Fig 3:**
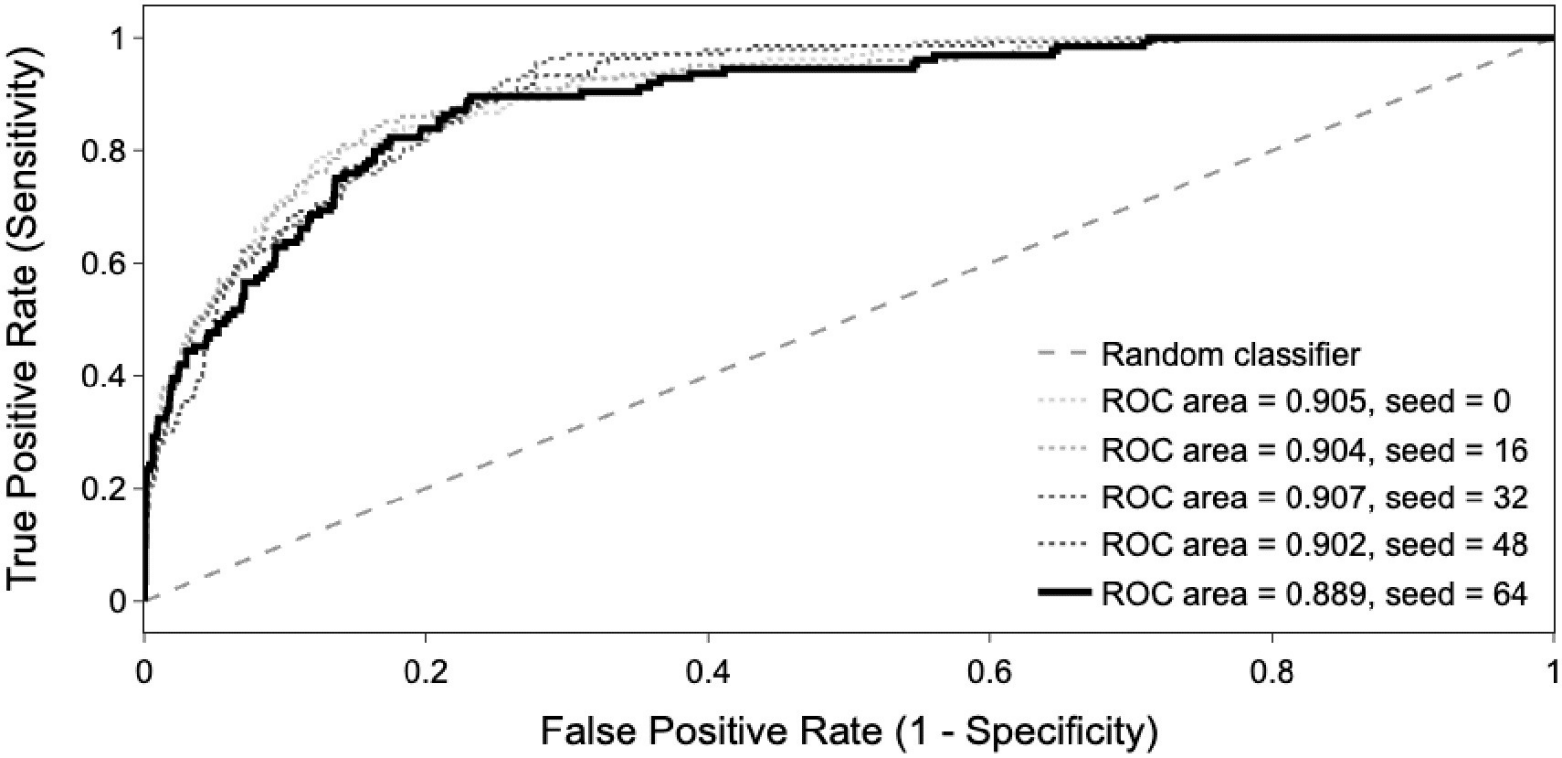
Performance metrics of logistic regression as a function of decision threshold. Indices of precision, recall, F1 score, accuracy. For example, for a threshold of 0.25, the F1 Score was 0.56, sensitivity 0.48, precision 0.86 and recall 0.68.

**Fig 4:**
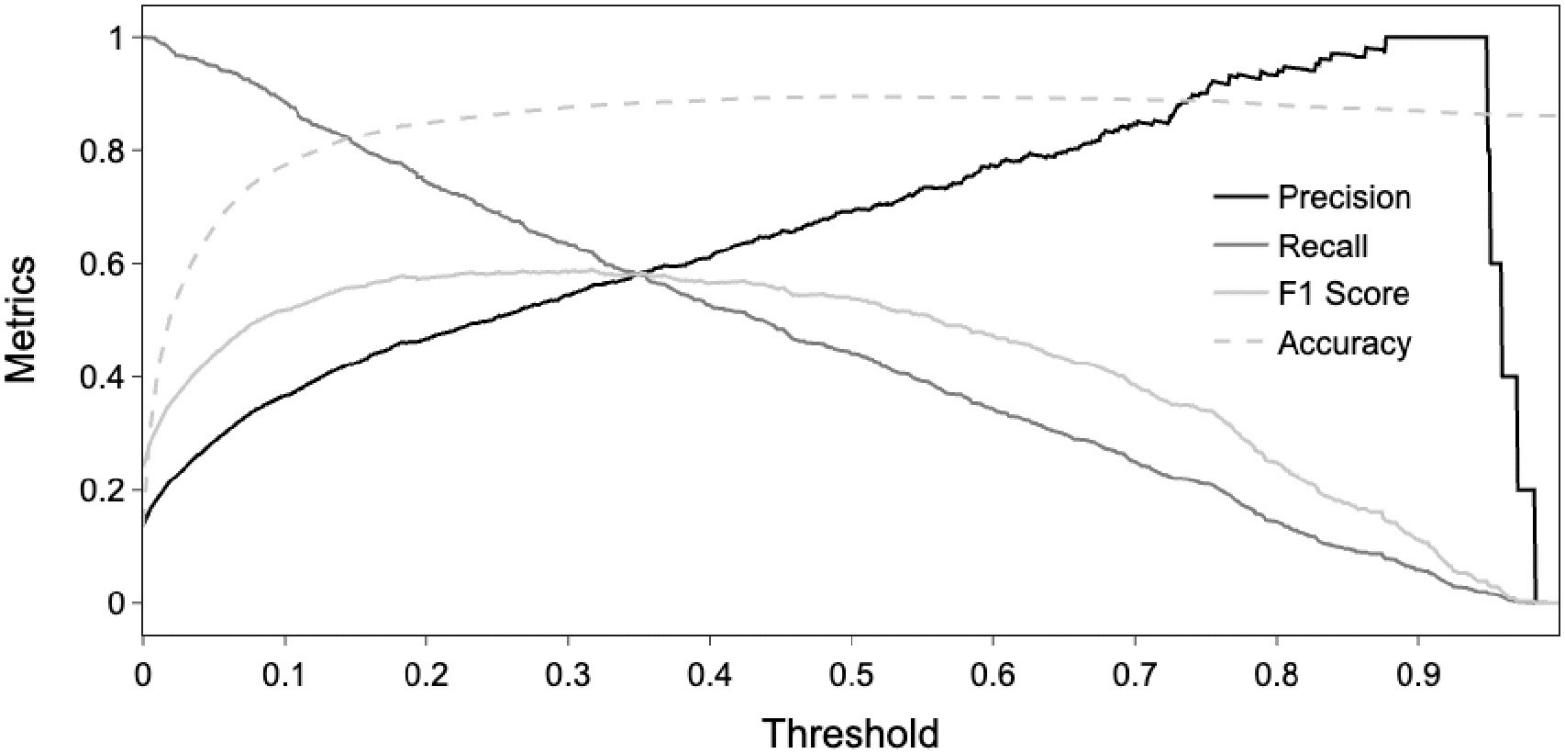
Receiver operator characteristic (ROC) curves from the logistic regression. The curve in bold is the one for seed 64, on which all other results presented are based. For a threshold of 0.18, the ROC area was 0.89, F1-score 0.57, recall 0.75, precision 0.46 and accuracy 0.85.

The accuracy of the logistic regression prediction model was assessed on the test set Fig 3. Fig 4 gives the ROC curves with the data stratification seed equal to 64, as well as for 4 other seeds. The areas under each of these curves (AUC) are in the range 0.89–0.91. Table 2a shows the coefficients of the logistic regression for the different variables together with SD, 95% CI, Z-statistic and P-value (with the data stratification equal to 64).

**Table 2:** a. The nine of most relevant variables (among the initial 12), in standardized form obtained through exhaustive feature selection and five-fold cross-validation on the training set. b. Mean and standard deviation of the most relevant variables, in unstandardized form.

**Variables**	**B**	**SD**	**Z**	**P**	**95% CI**	**e^B**	**95% CI**
**Intercept**	-3.26	0.11	-29.27	0.000	(-3.47:-3.04)	0.04	(0.03:0.05)
**Nationality**	-0.38	0.05	-7.91	0.000	(-0.47:-0.28)	0.69	(0.63:0.75)
**Comp Type**	0.20	0.07	2.76	0.006	(0.06:0.34)	1.22	(1.06:1.41)
**Nb Comp**	0.31	0.09	3.16	0.002	(0.12:0.51)	1.36	(1.12:1.66)
**Nb Prog**	0.37	0.09	4.19	0.000	(0.20:0.54)	1.44	(1.22:1.72)
**Max Prog**	0.49	0.08	6.12	0.000	(0.33:0.65)	1.64	(1.39:1.91)
**Age Max Prog**	-0.23	0.06	-3.62	0.000	(-0.35:-0.10)	0.79	(0.70:0.90)
**Nb Reg**	-0.51	0.09	-5.35	0.000	(-0.70:-0.32)	0.60	(0.49:0.72)
**P2**	1.47	0.11	13.04	0.000	(1.25:1.69)	4.35	(3.48:5.42)
**P6**	0.53	0.10	5.31	0.000	(0.33: 0.73)	1.70	(1.39: 2.07)
**Variables**			**Mean**			**Standard Deviation**	
**Nationality**			0.9			0.3	
**Comp Type**			0.7			0.5	
**Nb Comp**			2.1			1.3	
**Nb Prog**			3.1			1.9	
**Max Prog**			70.2			18.6	
**Age Max Prog**			19.2			2.9	
**Nb Reg**			1.4			1.5	
**P2**			0.96			0.02	
**P6**			0.96			0.02	

Corresponding logistic regression coefficients (B), standard deviation (SD), Z-statistics, P-value, 95% CIs, odd, e^B, 95% CIs for e^B obtained through a refit on the entire training set.

For the variable number of career progressions, the average all performances were 3.1±1.9 progressions and for the variable age at maximum progression (Age Max Prog) the average for the whole population was 19.2±2.9 years.

### Relative strength of the variables

From Table 2a, we first observe that the odds of winning a medal over all performances are about 1:25 (e^B = 0.04). Six of the nine variables influence the odds positively. By comparing the coefficients of the standardized variables (Table 2a), we see that the strongest variable is P2, then come variables P6, max_prog, nb_prog, nb_comp (number of competitions) with comparable strengths, and finally comp_type. Three of the nine variables influence the odds negatively: nb_reg, nationality, and age_max_prog, with the latter being somewhat weaker than the other two.

### Interpretation of the coefficients

An interpretation of the coefficients can be obtained by considering the increase or the decrease of the odds as the unstandardized variables vary (see Table 2b). If a variable X is increased by Δ, the odds of winning a medal gets multiplied by e^BΔ/std^, where B and std denote the coefficient and the standard deviation of X, respectively. From Table 2a and 2b we obtain:

- Nationality: non-US athletes have their odds of winning a medal multiplied by a factor between 0.2 and 0.37 (95% CI), i.e., the odds of winning a medal is reduced by 63–80% with respect to US athletes.
- Comp_type: participating in an OG multiplies the odds by a factor between 1.1 and 2.1 with respect to WC.
- Nb_comp (avg. ~2): Adding one more competition increases the odds of winning a medal by a factor between 1.1 and 1.5.
- Nb_prog (avg. ~3, std. ~2): Increasing the number of progressions by 1 multiplies the odds of winning a medal by a factor between 1.2 and 1.8.
- Max_prog: (avg. ~70, std. ~20): Increasing the number of progressions by 20 multiplies the odds of winning a medal by a factor between 1.1 and 1.2.
- Age_max_prog (avg. ~18, std. ~3): A decrease in the age of maximum progression by 3 years multiplies the odds of winning a medal by a factor between 0.7 and 0.9.
- Nb_reg (avg. ~1.5, std. ~1.5): An decrease in the number of regressions by 2 multiplies the odds of winning a medal by a factor between 0.4 and 0.6.
- P2 (avg. ~0.96, std. ~0.02): improving one’s best time two months before a competition by about 1% multiplies the odds of winning a medal by a factor between 1.8 and 2.5.
- P6 (avg. ~0.96, std. ~0.02): improving one’s best time in the two to 6 months period before a competition by about 1% multiplies the odds of winning a medal by a factor between 1.2 and 1.5.

## Discussion

The variables that predicted winning an Olympic swimming medal were nationality, a high maximum progression in the world rankings at a young age, a high number of progression phases and a low number of regression phases, a high number of competitions, and finally a high level of performance 6 and 2 months before the competitions relative to the previous year. We observe that the excellent value of the AUC (aggregate measure of model performance) is robust with respect to the random data stratification; the five AUCs are all in the range 0.89–0.91. A high AUC highlights the high predictability of winning medals in international swimming competitions, from the 9 predictive variables (7 performance variables and 2 non-performance).

Our results showed a higher probability of winning an Olympic or World medal for American swimmers compared to other swimmers. The odds of winning a medal for non-US swimmers are reduced by 63–80% compared to US swimmers. The US has been the strongest nation in swimming for decades, and has won about 40% of the medals at the OG and WC. The US likely combines a favorable combination of factors related to athletic excellence (i.e., large population, strong economic power, diverse athletic culture, national coaching expertise, and well-developed infrastructure) [24,25,27]. In second place for the number of Olympic medals since 1984, Australia, with 15% of the medals at stake, is demographically and economically substantially smaller than the USA, but has a sporting cultural tradition and organizational excellence, as do nations such as Norway in the winter disciplines [24], sprinting in Jamaica [25] and sailing in Denmark [26]. Over the study period eight other nations won between 4% and 8% of the medals: China, German Democratic Republic, Hungary, Japan, Great Britain, Germany, Russia and Italy. The competitiveness of these nations was variable throughout the study period likely due to fluctuations in their organizational effectiveness (institutional structures and support programs) and cultural and technical developments (i.e., training methods, technological materials, values and beliefs conveyed by previous successes) [24,28–30].

### Seasonal progressions

Swimmers progression in world rankings from one season to the next was associated with the probability of winning a medal. Over the total length of a swimmer’s career, further progress in a season compared to the previous one increases the odds of winning a medal by a factor between 1.2 and 1.8 and each 20-place increase in the world ranking multiplies the odds of winning a medal by a factor of between 1.2 and 1.8. For example, in the 100 m and 200 m freestyle, 20 places in the world ranking (the difference between first and twentieth) represents 94 hundredths and 1 second and 44 hundredths respectively. Several previous studies in swimming [1], athletics [4,16,17] and cross-country skiing [5]. have already reported a higher rate of multi-year improvement in performance among elite athletes when compared to lower-level athletes. Costa and colleagues [2010] analyzed the annual progressions of 477 swimmers over five competitive seasons (2385 seasons in total) highlighting that Olympic medal-winning athletes improved their performances by ~0.6 to 1% between seasons and 3 to 4% over the entire Olympic cycle. This cohort showed a greater progression than the 2.4 ±1.2% observed over four years in non-medal-winning Olympic qualified swimmers [1,2]. Although the world’s top athletes show similar pre-pubertal performance progress early in their careers as other lower-level athletes, they typically accelerate their progress during and after puberty [4,5,14–17]. Analysis of the performance progressions of 4076 swimmers who participated in the junior and senior WC, showed the annual performance improvements were greater for the 30% of swimmers with the best performances [19]. In the same vein as previous research [1,2,19,21], our results confirm a strong progression of a swimmer in the world ranking is associated with a higher probability of winning a WC or OG medal. Significant progression of Olympic medalists likely depends on many interacting factors: more effective training to promote biological adaptations of athletes [Georgiades, 2017], greater phenotypic plasticity of champions [4,5,31] that can operate as a "Matthew effect" [32], strong support from families, coaches and networks [4–7,26,27], and a sociocultural and organizational environment oriented towards success in elite sport [4–7,26,27].

Our results indicated that the younger age the maximum progression in world rankings was achieved the higher the probability of being a medalist. Decreasing the age of maximum progression by 3 years increases the odds of winning a medal by a factor of between 0.7 and 0.9. In agreement, Haugen and colleagues [4] have shown that athletes need to be at a very high level from their late teens to become world-class athletes. However, other work has shown that early pre-pubertal success (before age 12) was only weakly associated with success at the highest world-level [33]. Participation in numerous junior competitions and within-sport diversification in swimming are explanatory factors for achieving athletic excellence at the senior level [34–36]. It further appears that years of high-level competition have a positive impact in achieving better positions during the senior WC [35]. Our results complement previous research [13] highlighting that the higher the number of competitions performed by swimmers, the higher the probability of achieving a medal at the World or Olympic level. The number of races completed and the number of years of participation in the junior world championships were moderately related to the results of the European championships [13] and senior WC [35]. In a highly complex sport such as swimming, multiple physiological, psychological (perceived control, mental toughness, resilience, coping with adversity, resistance, mental skills) and technical (biomechanical efficiency, starts, turns, race pace) elements are acquired and reinforced through regular exposure to competition.

### Effect of progressions and regressions

The probability of winning a medal was associated with more progressions in performance, and fewer regressions, throughout the swimmers’ career. This factor has a strong impact on the probability of winning a medal because increasing the number of regressions by 2 multiplies the odds of winning a medal by a factor between 0.4 and 0.6. Tracking career performance trajectories has revealed nonlinear evolutions with highly variable oscillations [17,37,38]. These research’s however, did not link these performance fluctuations to success at the world level. Our research is the first to link a high number of progressions and a low number of regressions to a higher probability of winning WC or OG medals. This result means that medalists are better able to maintain their annual performance level even in years with less competitive deadlines (i.e. European Championships or Pan Pacific Games). Achieving annual progression throughout careers of 5–15 years in duration likely depends on multiple factors related to the athlete, sport ecosystem, and training [7,31,38]. Case studies of multiple world medal-winning athletes show a progressivity of training loads throughout the career that often correlates with an increase in physiological capacity until the age of 30 y [39,40]. In addition to training progressivity, targeted exposure to specific impactful training stimuli (hypoxia, high-intensity training) mobilizes reserves of potential progressions and promotes continued improvement in performance [40]. Moving out of an individual athlete’s comfort zone to continue to progress once the performance plateau is reached requires coping with fatigue induced by multi-year training loads. This scenario likely requires high levels of motivation, self-confidence, stress resistance, and resilience [7,13]. Moreover, champions are characterized by a total qualitative commitment over the long term [41], with elements of obsession, perfectionism, ruthlessness and a dual focus on mastery and results [42]. Finally, Olympic medalists know how to transcend themselves to perform at their highest level on ‘D-day at H-hour’ [22]. These characteristics of success presumably evolve over time through exposure, competition and self-reflection.

The link between the number of annual regressions and a lower probability of winning a WC or OG medal is a novel finding compared to previous research [1,15–17]. In our study, this variable had the third strongest impact on the probability of winning a medal. Previous research [4,5,15–17,37] did not observe clear differences in annual progressions and regressions between Olympic and lower-level athletes [37]. From the peak and plateau of performance estimated respectively at 24 ± 2 years and 26 ± 0.5 years [1], studies describe a progressive decline in performance. A variety of explanations were proffered: a slow decline in physiological potential, misunderstanding with the trainers, academic and socio-professional demands, failure or injury, family or institutional pressure, and/or lack of enjoyment [37]. Our results in the current study commit institutions and coaches to developing and implementing programs and strategies to counteract the potential negative events affecting athletes throughout their career.

### Performances prior to international competition

Improving performance by 1% in the two to six-month period prior to the major competition (WC or OG) increases the odds of winning a medal by a factor of between 1.2 and 1.5, while a 1% improvement in the two-month window increases the odds by a factor of between 1.8 and 2.5. P2 and P6 are the two factors with the greatest impact on the probability of winning a medal at the World Championships or Olympic Games. The probability of winning a medal is likely linked to a progressive rise in performance throughout the season as already suggested by other authors [20,21]. From 7832 official competition times recorded at 4 FINA World Championships and 2 Olympic Games between 2011 and 2017 performance improvement between the selection event and the Olympic Games was greater for medal winners (-0.87 ± 0.91%) than finalists (-0.16 ± 0.97%), semi-finalists (0.21 ± 0.97%) and swimmers who did not make it through the heats (0.90 ± 1.16%) [21]. To remain in contention for a medal, an Olympic swimmer must improve their performance by ~1% in the year prior to the Olympic Games and beat his or her personal best performance by approximately 0.35% [19]. Our study is the first to suggest performance thresholds below which the probability of being a medalist decreases. For instance, in both the six-month and two-month windows (Fig 2a and 2b), at least 50% of gold/silver/bronze medalists achieved performances equal to or greater than 98% of the previous year’s world best. Conversely, less than 20% of gold medals were associated with a P2 and P6 of less than 97%.

### Practical applications

Federations and coaches should address all factors that promote the greatest progression at the world level, increase the number of progressions and reduce the number of regressions in performance. Coaches should periodize the training load blocks and associated recovery to progressively improve performance in the 6-to 2-month window prior to major competitions in order to peak at the highest possible level. Predictions can provide decision support for team selection at competitions, and strategic decisions regarding resource allocation. The logistic regression model we established yields an AUC of ~0.9, which corresponds to assigning a higher score to a medalist than to a non-medalist with ~90% probability.

### Strengths, limitations and future research

Methodologically, our research on multi-year performance evolution and critical factors for swimming performance is the first to adapt machine learning procedures using a logistic regression model in swimming. In the majority of previous research, the models used mean exponential growth [14], multiple linear regression [17], and individual quadratic curves via mixed modeling [1–6,13]. In these studies, the predictions were made from the models fitted on the initial dataset, and not from cross validation procedures on unknown test data set that permit more robust predictions [43]. Thus, this research presented in this study is both comprehensive and highly predictive of competitive swimming performance. The AUC on the prediction data set indicates that the model is able to detect 89% of the medalists for future swimming competitions not considered for the model fitting. Modifications of the decision threshold of the classifier represented in Fig 3 can be used to adjust the ratio between detection of true positives (medalists detected as medalists) and false positives (non-medalists detected as medalists). However, the interpretation of the coefficients should be taken with caution given the likelihood of potential dependencies. Finally, in athletics [4] and cross-country skiing [5], two studies showed that the age at peak performance and the percentage of progression during the 5 years preceding peak performance differed depending on the event (sprint, middle-distance, cross-country running, throwing). Also, future study may examine the different predictors of success in different sprint and middle-distance events, and possibly freestyle, backstroke, breaststroke and individual medley events.

## Conclusion

The likelihood of winning medals at the World Championships and Olympic Games is associated with nationality, a strong progression in the world ranking achieved at a young age, completing a high number of competitions developing technical, physical and mental skills and more progressions and fewer regressions throughout the career. In the season leading up to the major international competition(s), the higher the performances relative to the world’s best performance and progression in the 6- to 2-month window, the higher the probability of winning a medal. Coaches, support staff, and swimming organizations can use these outcomes to inform training and athlete development programs.

*The results of this study are presented clearly, honestly, and without falsification, or inappropriate data manipulation. The results of the present study do not constitute endorsement by the American College of Sports Medicine*.
